# Heart irradiation reduces microvascular density and accumulation of HSPA1 in mice

**DOI:** 10.1007/s00066-017-1220-z

**Published:** 2017-10-23

**Authors:** Anna Walaszczyk, Katarzyna Szołtysek, Karol Jelonek, Joanna Polańska, Wolfgang Dörr, Julia Haagen, Piotr Widłak, Dorota Gabryś

**Affiliations:** 1 Center for Translational Research and Molecular Biology of Cancer, Maria Skłodowska–Curie Memorial Cancer Center and Institute of Oncology Gliwice Branch, Wybrzeże Armii Krajowej 15, 44-101 Gliwice, Poland; 2 Silesian University of Technology, Gliwice, Poland; 3 Department of Radiotherapy and Radiooncology, Medical Faculty Carl Gustav Carus, University of Technology, Dresden, Germany; 4 Department of Radiation Oncology, Applied and Translational Radiobiology (ATRAB), Medical University Vienna, Vienna, Austria; 5 Department of Radiotherapy, Maria Skłodowska–Curie Memorial Cancer Center and Institute of Oncology Gliwice Branch, Wybrzeże Armii Krajowej 15, 44-101 Gliwice, Poland

**Keywords:** Cardiotoxicity, Adverse effects, Cardiovascular system, HSPA70-1 heat-shock proteins, Myocardial ischemia, Kardiotoxizität, Nebenwirkungen, Kardiovaskuläres System, HSPA70-1 Hitzeschockproteine, Myokardiale Ischämie

## Abstract

**Purpose:**

Improvement of radiotherapy techniques reduces the exposure of normal tissues to ionizing radiation. However, the risk of radiation-related late effects remains elevated. In the present study, we investigated long-term effects of radiation on heart muscle morphology.

**Materials and methods:**

We established a mouse model to study microvascular density (MVD), deposition of collagen fibers, and changes in accumulation of heat shock 70 kDa protein 1 (HSPA1) in irradiated heart tissue. Hearts of C57BL/6 mice received a single dose of X‑ray radiation in the range 0.2–16 Gy. Analyses were performed 20, 40, and 60 weeks after irradiation.

**Results:**

Reduction in MD was revealed as a long-term effect observed 20–60 weeks after irradiation. Moreover, a significant and dose-dependent increase in accumulation of HSPA1, both cytoplasmic and nuclear, was observed in heart tissues collected 20 weeks after irradiation. We also noticed an increase in collagen deposition in hearts treated with higher doses.

**Conclusions:**

This study shows that some changes induced by radiation in the heart tissue, such as reduction in microvessel density, increase in collagen deposition, and accumulation of HSPA1, are observed as long-term effects which might be associated with late radiation cardiotoxicity.

**Electronic supplementary material:**

The online version of this article (10.1007/s00066-017-1220-z) contains supplementary material, which is available to authorized users.

## Zusammenfassung

**Zielsetzung:**

Die Verbesserung der Strahlentherapietechnik reduziert die Exposition von normalen Geweben mit ionisierender Strahlung. Allerdings bleibt das Risiko strahlenbedingter Spätfolgen erhöht. In der vorliegenden Studie untersuchten wir die Langzeitwirkung einer Strahlenexposition des Herzmuskels in Bezug auf morphologische Veränderungen.

**Material und Methoden:**

Wir haben ein Mausmodell etabliert, um die mikrovaskuläre Dichte (MVD), Ablagerung von Kollagenfasern und Veränderungen der Akkumulation von 70kDa-Hitzeschockprotein 1 (HSPA1) in bestrahltem Herzgewebe zu untersuchen. Männliche C57BL/6-Mäuse erhielten in Einzeldosen Röntgenstrahlen zwischen 0,2–16 Gy. Die Herzen wurden für die Analyse 20, 40 und 60 Wochen nach der Bestrahlung entnommen.

**Ergebnisse:**

Als Langzeitwirkung 20–60 Wochen nach der Bestrahlung zeigte sich eine Reduktion der MVD. Weiterhin zeigte sich 20 Wochen nach Bestrahlung eine signifikante und dosisabhängige Akkumulationszunahme von HSPA1, sowohl zytoplasmatisch als auch nukleär. Auch wurde eine Kollagenablagerung in Herzgeweben festgestellt, die mit höheren Dosen behandelt wurden.

**Schlussfolgerung:**

Diese Studie zeigt, dass die Bestrahlung zu Veränderungen, wie Verringerung der MVD, Zunahme der Kollagenablagerung und Akkumulation von HSPA1 im Herzgewebe, führt, die mit einer späten Strahlenkardiotoxizität assoziiert sein können.

Cancer and heart diseases are the major cause of death worldwide. Over 50% of cancer patients are treated with radiotherapy. Modern radiotherapy techniques applied in recent decades contribute to increasing long-term survival of cancer patients. However, long-term effects of successful anticancer treatment involve an increase in late adverse events. Radiotherapy, if applied in the thoracic region, represents a potential risk factor for the cardiovascular system. Cardiovascular damage has been reported as a long-term adverse/side effect event in breast cancer survivors previously treated with radiotherapy. The risk of cardiovascular diseases is correlated with tumor location and was found to be significantly increased following irradiation of the left breast compared to the right and the rate of ischemic heart disease was significantly increased, even with doses <2 Gy [1–4]. Therefore, experimental animal and cellular studies are necessary, not only for a correct extrapolation of risk estimations, but also for the evaluation of biological mechanisms identification of potential biomarkers of the risk, and the development of therapeutic countermeasures [5].

In order to explore mechanisms behind the pathogenesis of early and late radiation-related alterations in the microcirculation of the heart, the European Consortium called CARDIORISK was formed in 2008 and the following work was performed within its framework. This project addressed both macro- and microvascular radiation effects after local irradiation of the heart, with a special focus on the radiation response in the low-dose range [6].

Radiation-induced heart disease includes a wide spectrum of cardiac pathologies, including pericarditis, cardiomyopathy, myocardial fibrosis, valvular disorders, coronary artery disease, and conduction abnormalities. Factors enhancing the risk of cardiotoxic effects of radiation are both treatment-related and endogenous. On the list related to treatment are the following: high dose, large irradiated volume, concomitant chemotherapy—especially the use of anthracyclines. Among endogenous factors increasing risk of treatment side effects are the following: young age of patients, overweight, atherosclerosis, and hypertension [7, 8]. Endothelial cells are the primary target for ionizing radiation across the myocardial vasculature. The main acute effect of radiation is accumulation of lymphocytes and as a consequence inflammation. Inflammation induces contraction of blood vessels in the heart muscle. This may lead to remodeling of the cytoskeleton and formation of gaps between adjacent endothelial cells [9]. Myocardial ischemia is dominant among late effects and is usually accompanied with increased collagen synthesis and fibrosis [10, 11].

The 70-kDa heat shock protein (HSP70) family constitutes one of the most conserved protein families [12, 13]. HSPA1 (also known as HSP70-1, HSP72, or HSP70i) is the main stress-induced HSP and among the first and the most prominent proteins which are found in stressed cells. Therefore, this protein is often used as a general marker of a stress response [14]. Heat shock proteins (HSPs) represent chaperones that protect cells against a variety of stresses. They are involved in the regulation of the immune responses including antigen processing and cross-presentation [14]. Expression and accumulation of heat shock proteins in heart tissue could be induced by inflammation, hypoxia, chemotherapy, hyperthermia, and oxidative stress [15–20]. Stress-induced upregulation of HSPs promotes cell (including cardiomyocytes) survival [12, 21]. Although HSP are widely analyzed, we still lack information regarding HSP changes in the heart after exposure to ionizing radiation.

Currently, only several radiobiological models relevant for assessment of cardiotoxic effects of ionizing radiation are available. One of the models that considers the complex and long-term nature of radiotherapy-related damage involved in heart failure uses mouse hearts from animals irradiated in vivo. In the present study, we investigated long-term effects of radiation on heart muscle morphology. We analyzed microvascular density (MVD), deposition of collagen fibers, and changes in accumulation of stress-inducible heat shock 70 kDa protein 1 (HSPA1) in irradiated mouse heart tissue.

## Materials and methods

### Animals and irradiation procedure

The experimental procedures have been reported in detail elsewhere [22]. In brief, male C57BL/6 mice (Charles River Laboratories, Research Models and Services, Germany GmbH) aged 8 ± 1 weeks were immobilized and locally irradiated using a YXLON MG325 device (Yxlon International X‑ray GmbH, Germany) operated at 200 kV, with a tube current of 20 mA and a beam filter of 0.6 cm Cu, resulting in a dose rate of 0.8045 Gy⋅min^−1^. Single local heart doses of 0.2, 2, 8, or 16 Gy were applied. The exact position of the heart was assessed by radiography before irradiation. The whole heart was included in the irradiated volume, plus about 20% of the lung, but excluded the liver and other organs, as previously reported [23]. Age-matched, sham irradiated (0 Gy) animals were included in the study. Animals were irradiated in Dresden, shipped to Gliwice 1–2 months after irradiation and maintained on a regular chow diet (1324 TPF standard diets, Spezialfutter GmbH & Co. KG, Germany) and tap water ad libitum. The environmental conditions were as follows: temperature of 21 ± 2 °C, humidity of 55 ± 10%, illuminance of 350 lx (at bench level) and a 12:12 light: dark cycle. Animals were housed in 207 × 140 × 365 mm cages (type IIL, BIOSCAPE, Germany). Each treatment group comprised 5 mice.

### Tissue preparation

After 20, 40, and 60 weeks following irradiation the animals were sacrificed by cervical dislocation, the hearts were excised, divided into 3 pieces on the horizontal axis (apex, ventricles, and atria). From each heart, the ventricles were cryoembedded for immunohistochemistry, the apex was frozen for gene expression analysis, and the atria were fixed with 37% formaldehyde. For analysis of ultrastructure using electron microscopy, a small piece of the left ventricle was fixed in buffered 3% glutaraldehyde in cacodylate buffer (unpublished results). Cross-sections of the ventricles were cut in a horizontal plane (7 µm). Of these sections, 5 to 6 were analyzed per heart, each representing a different part of the ventricle. The average number of images analyzed per heart was 23. In total, we analyzed about 600 images to evaluate microvessel density. To analyze collagen fiber deposition, we established a similar pattern. In total, about 470 images were analyzed. To evaluate the HSPA1, we analyzed the positive area (estimated as a percentage of whole tissue section); two complete cross-sections were inspected for each mouse and several images were analyzed to cover each cross-section. The protocol was approved by the Committee on the Ethics of Animal Experiments of Landesdirektion Dresden (file no. 24-1968.1–11/2009-10; Germany).

### Immunohistochemistry

An anti-CD31 antibody (1:50, Becton and Dickinson) was used to visualize cardiac vasculature in the ventricles. An anti-HSP70i antibody (1:50, Stressgen SPA-810) was used to evaluate the expression of HSPA1 protein. The frozen sections were air-dried. Endogenous peroxidase activity was blocked with 0.3% hydrogen peroxide in methanol for 30 min. Endogenous biotin was suppressed with the avidin/biotin kit (15 min each; DAKO, ITK Diagnostics BV, Uithoorn, The Netherlands). Protein block serum-free (DAKO) was used to minimize unspecific binding of the antibody (20 min), then slides were incubated overnight with primary antibodies (diluted 1:50 with 1% bovine serum albumin [BSA]/phosphate-buffered saline [PBS]) at 4 °C. The slides were then rinsed with PBS and subsequently incubated with biotinylated secondary antibodies (45 min). After rising with PBS, the signal was amplified with avidin–biotin complex (ABC) reagent (Vectastain ABC Kit). Antibody reactivity was detected using horse-radish peroxidase (HRP)-conjugated biotin–avidin complexes (Vector Laboratories, Burlingame, CA, USA) and developed with diaminobenzidine (DAB) solution (Sigma). All sections were processed identically within one experiment. For microvascular density analysis tissue slides were photographed with Nikon Eclipse 80i, 40 × objective, five random fields per slide were taken into consideration.

### Collagen deposition

The trichrome method was used for detection of collagen fibers in the heart muscle preparations as described by Gomori [24]. Using this method, cell nuclei are stained in dark blue or black, muscle fibers are stained in red, while the connective tissue, primarily collagen, is presented in green. Based on Gomorie trichrome staining, collagen was determined in heart ventricles. Slides were fixed with 37% formaldehyde, incubated with Bouin’s reagent (Sigma-Aldrich HT-10-1-128, 15 min, 56 °C,) and, after washing with tap water, stained with Weigert’s iron hematoxylin (Sigma-Aldrich HT10-7, HT10-9). After that, slides were stained with LG (Gomori one-step trichrome [light green] procedure) reagent (Sigma-Aldrich HT10-3-16) then washed for 1 min in 0.5% acetic acid, dehydrated (50–100% ethyl alcohol) and mounted in DPX mounting medium for histology (Sigma-Aldrich). Slides were analyzed with Nicon Eclipse 80i, 20 × objective; five random fields were photographed per slide.

### Statistical analysis

All technical repeats (average 23 replicates) were combined for each experimental group. The Shapiro–Wilk test was applied to verify the hypotheses on normality of distribution across experimental groups [25]. Homogeneity of dispersion was tested by Brown–Forsythe test [26]. Equality of median values was checked with the use of Mann–Whitney U test [27]. Hodges–Lehmann minimum distance estimator and its 95% confidence interval (CI) were used for estimation of the shift between distributions [28]. Results with *p* < 0.05 were considered as statistically significant.

## Results

### Vessel density

**Fig. 1:**
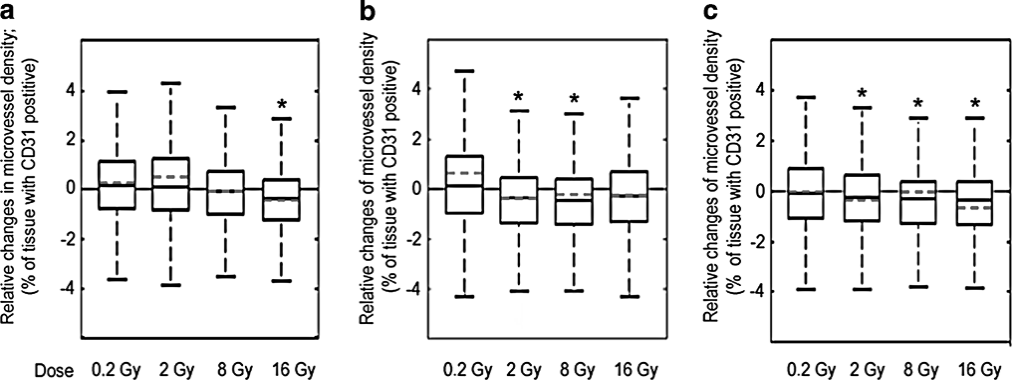
Changes in microvascular density. The figure illustrates relative changes in the microvascular density (% of area positive for anti-CD31; ratio irradiated animals and control) in hearts irradiated with doses ranging from 0.2–16 Gy, relative to age-matched not irradiated controls. Samples were analyzed at 20 (**a**), 40 (**b**), 60 (**c**) weeks after irradiation. Boxplots show maximum and minimum, upper and lower quartiles, and median; *horizontal dashed line* represents arithmetic average; *asterisks* indicate significant differences (*p* < 0.05) between control and irradiated animals

Staining for CD31, an antigen specific for endothelial cells, was used to estimate microvascular density (MVD). The results are presented in Fig. 1. In the group of animals analyzed 20 weeks after irradiation with 16 Gy, we observed a statistically significant decrease of MVD. In animals analyzed 40 weeks after irradiation, statistically significant reduction of MVD was observed after 2 and 8 Gy, while a similar trend was observed after 16 Gy (*p* = 0.055). In animals analyzed 60 weeks after irradiation, a statistically significant reduction of MVD was found after irradiation with 2, 8, and 16 Gy. In the animals irradiated with a low dose (0.2 Gy), no changes in microvascular density were observed at any follow-up period.

### Collagen deposition

**Fig. 2:**
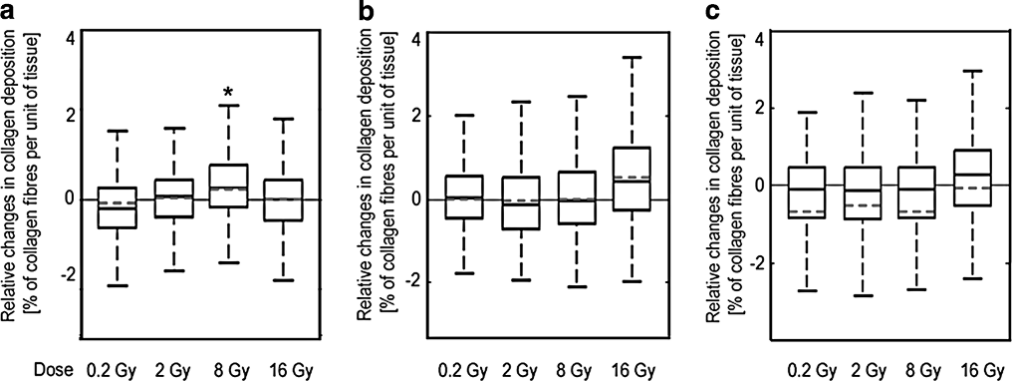
Collagen fiber deposition. The figure shows relative changes of tissue areas positive for trichrome staining (% of heart area; ratio irradiated animals and control). Samples were analyzed at 20 (**a**), 40 (**b**), or 60 (**c**) weeks after irradiation. Boxplots show maximum and minimum, upper and lower quartiles, and median; *horizontal dashed line* represents arithmetic average; *asterisks* indicate significant differences (*p* < 0.05) between control and irradiated animals

A statistically significant increase in collagen deposition was observed in heart tissue of mice analyzed 20 weeks after irradiation with 8 Gy. Moreover, a similar trend was observed for animals analyzed 40 and 60 weeks after irradiation with 16 Gy (Fig. 2).

### HSPA1 protein accumulation

**Fig. 3:**
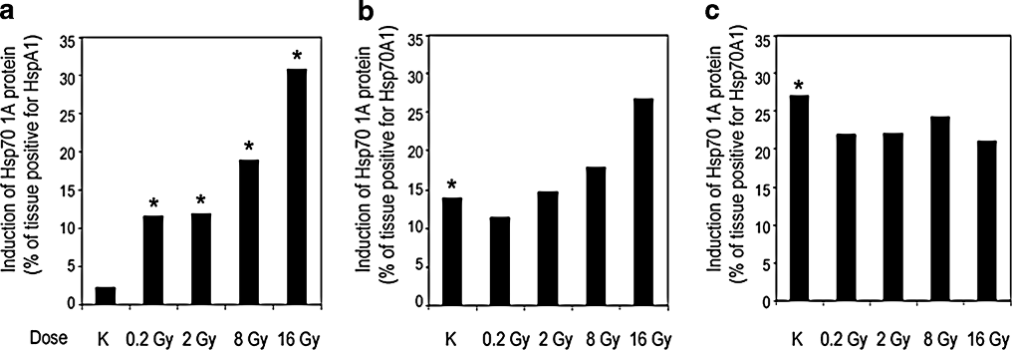
Changes in Hsp70A1 protein accumulation. The figure illustrates changes in the relative accumulation of HspA1 (% of area positive). Samples were analyzed 20 (**a**), 40 (**b**), or 60 (**c**) weeks after irradiation. *Bars* show the median value; *asterisks* indicate significant differences (*p* < 0.05) between irradiated animals and control (**a**), or between 48- and 68-week-old control animals with respect to control 28-week-old control mice (**b**, **c**)

A statistically significant dose-dependent increase in accumulation of HSPA1 (staining-positive area in relation to the control) was observed in heart tissues analyzed 20 weeks after irradiation (Fig. 3). At 40 weeks, there seems to be a trend for a dose response, although not significant. In contrast, 60 weeks after irradiation similar levels of HSPA1 were observed in both irradiated and control animals.

However, it should be noted that a clear age-related increase in HSPA1 protein levels was detected in nonirradiated control animals (Fig. 3). Moreover, cellular localization of HSPA1 was analyzed in hearts of animals exposed to radiation using a fluorescence-labeled anti-HSPA1 antibody and DAPI counterstaining (see Supplementary Fig. S1). We found expression of HSPA1 in both the cytoplasm and the nucleus at 20 weeks after exposure to 16 Gy. This was in marked contrast to cardiac cells of animals exposed to hyperthermia (unpublished data), where a primarily nuclear localization of HSPA1 was observed 18 h after the heat shock (Supp. Fig. S1).

## Discussion

Improvement of radiotherapy techniques reduces the exposure of normal tissues to ionizing radiation. In the case of the heart, it considerably reduces the risk of early cardiovascular complications [29]. However, the risk of late effects of radiation remains elevated even after decades [30]. Endothelial cells comprise only a minor fraction of cardiac cells, but they are critical for radiation-induced damage [31]. In our study, reduction in microvascular density was revealed as a long-term effect observed 20–60 weeks after irradiation. These observations are consistent with results obtained by other laboratories forming the CARDIORISK consortium. Gabriels et al. [32] showed reduction of MVD in a heart 20 and 40 weeks after irradiation of ApoE (−/−) mice with doses of 8 and 16 Gy. Seemann et al. [33] reported decrease in MVD after 40 and 60 weeks following irradiation with the doses of 8 or 16 Gy in C57/BL6 mice.

On the other hand, no clear differences were detected in the MVD 16 weeks after irradiation in C57BL/6 mice by Azimzadeh et al. [34]. Small quantitative differences between these data and our results are most likely a consequence of a different heart area subjected to analysis, or different staining or analytical procedures. In our study, the measurement of MVD was performed in the ventricles of the heart in a cross section, while the group of Seemann and Azimzadeh analyzed only the left ventricle. These types of heart tissue changes may induce or intensify an existing heart disease, which eventually may have an impact on the function of the heart [35]. In fact, our results and those from others confirm that ionizing radiation reduces vascular density irrespective of the subvolume of the heart analyzed and differences in treatment protocols.

The reduction in the density of vessels likely results from endothelial cell damage induced by ionizing radiation. As a consequence, local hypoxia may be induced, leading to damage of both small vessels and the myocardium. One of the effects of myocardial ischemia is progressive fibrosis [36, 37].

Therefore, one may expect progressive fibrosis especially in the case of high doses and after a long follow-up. However, our results do not confirm this hypothesis. Similar results were obtained in other analyses within the CARDIORISK consortium. After exposure to low doses of radiation (0.2 or 2 Gy), there was a significant increase in the density of collagen fibers only in ApoE (−/−), but not in C57BL/6 mice [23]. In another study, the presence of collagen was elevated for C57BL/6 mice after 40 weeks of exposure to 8 or 16 Gy. However, even 60 weeks after exposure to a dose of 2 or 8 Gy, these changes were only seen in a small volume (2–5%) of the heart [33]. Difference in the response between the two mice strains may be related to the fact that accumulation of lipid deposits caused by malfunction of lipid metabolism in ApoE^--/--^ mice leads to oxidative processes, which could be intensified by irradiation, causing significant collagen formation. Perhaps in wild-type mice, irradiation without an additional risk factor is not sufficient to cause increased collagen deposition.

In the present study, involvement of HSPA1 in long-term effects of heart irradiation was documented. Accumulation of HSPA1 protein was increased 20 weeks after irradiation in a dose-dependent manner. Longer follow-up (40 or 60 weeks after irradiation) revealed increased HSPA1 protein accumulation in both control and irradiated animals, which indicates that changes induced by radiation per se may not be detected in older animals. Actually, it is known that aging induces structural and functional changes in the heart and vasculature. Furthermore, in elderly individuals, cardiac mechanisms responsible for protection from injury as well as injury repair itself become increasingly defective, thus, leading to accentuated adverse remodeling and increased dysfunction (reviewed in [38]).

It is noteworthy that HSPA1 accumulated in response to ionizing radiation showed ubiquitous cellular localization (both nuclear and cytoplasmic), which was in contrast to nuclear localization of this protein accumulated in “acute” response to hyperthermia.

Several lines of evidence indicated a role of HSPA1 in response to ionizing radiation and heart damage. In general, overexpression of HSPA1 is radioprotective and increases long-term viability (reviewed in [39]). Increased expression of HSPA1 was observed in endothelial cells isolated from C57BL/6 mice 16 weeks after X‑ray irradiation, which was accompanied by increased levels of oxidized low density lipoprotein (oxLDL) and impairment of NO production [34]. Moreover, expression of HSPA1 increase during cardiac hypertrophy in mice, and its overexpression protects the heart against damaging effects of ischemia [40, 41]. In humans with heart failure, plasma concentrations of HSPA1 increase gradually with the progression of disease stages; therefore, HSPA1 could be used as a potential screening biomarker for the early diagnosis of heart failure [40]. A plethora of HSPA1 functions involves activation of proinflammatory reaction or decreasing it in the cases of over-activation of the immune system. Thus, in the context of “oxi-inflamm-aging”, HSPA1 was proposed as one of the possible regulators of this process (reviewed in [42]). In humans, the serum concentration of HSPA1 decreases with age in a normal population while increased serum levels are frequently associated with frail health and inflammation [43].

In ICR-CD1 and Balb/C female mice, the 120-week-old group showed higher HSPA1 levels in the heart than the 82-week-old group, and these levels were similar to those found in 40-week-old animals [44]. Here we observed markedly higher level of HSPA1 in 48- to 68-week-old C57BL/6 male mice in comparison to 28-week-old animals (Fig. 3). The results of these studies cannot be compared directly because of differences in strain, age, and sex of the animals (ICR/CD-1 are more prone to inflammation than C57BL/6 mice, and the expression of cardiac HSPA1 could be different in female and male mice due to variable levels of estrogen [45, 46]). Nevertheless, both reports documented age-related increase in the cardiac HSPA1 level. Elevated levels of HSPA1 were found in human myocardial tissues in case of ischemia, following coronary artery bypass grafting or aortic cross-clamp surgery, and in patients with coronary artery disease (a protective role of HSPA1 has been suggested) [47]. In general, because HSPs have been considered as a potential therapeutic target for cardiac disease, it is important to further assess the role of HSPA1 in acute and chronic cardiac disease [48].

## Conclusion

The present study shows that some of the changes induced by radiation in the heart tissue, such as a reduction in microvessel density, increased collagen deposition, and accumulation of HSPA1, are observed as long-term effects which might be associated with late radiation cardiotoxicity. Better knowledge of the effects of radiation and the mechanisms of its formation may allow us to more effectively fight its cardiovascular consequences.

## Caption Electronic Supplementary Material

Fig. S1 Cellular localization of heat shock 70 kDa protein 1 (*HSPA1*) in heart of mice 20 weeks after exposure to 16 Gy and 18 h after 1 h heat shock at 43 °C. Tissue was stained with FITC-labeled anti-HSPA1 antibody and counterstained with 4’,6-diamidino-2-phenylindole (*DAPI*) to visualize nuclei (representative pictures were registered at 100 × magnification). *FITC* fluorescein isothiocyanate
