# Supplementary figures and images for: Heart irradiation reduces microvascular density and accumulation of HSPA1 in mice
Source: Strahlenther Onkol. 2017 Oct 23;194(3):235–42. doi: 10.1007/s00066-017-1220-z (PMC5847036; doi:10.1007/s00066-017-1220-z)

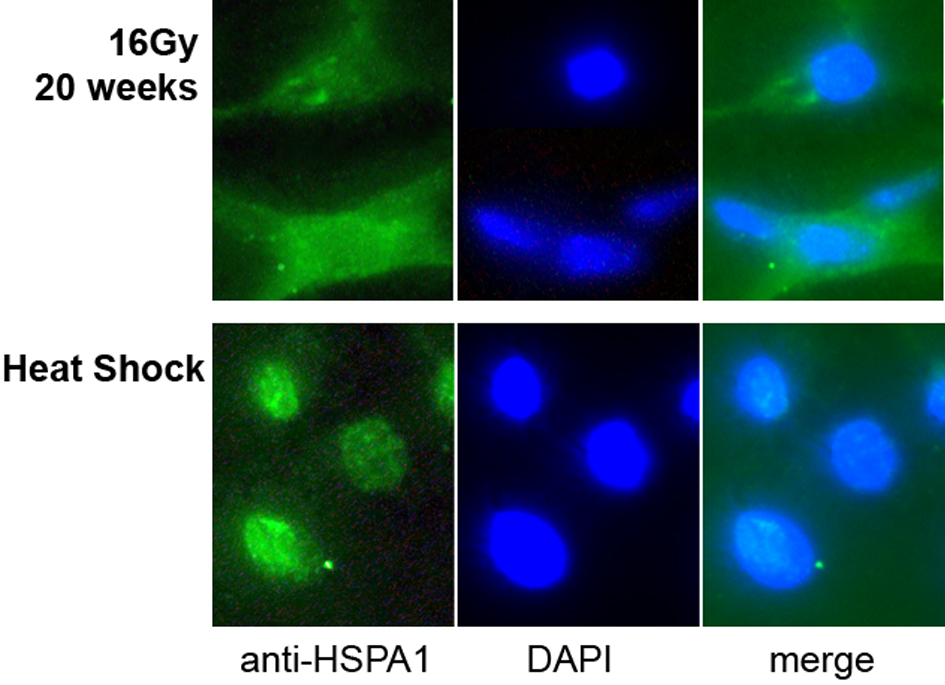

Supplement: Supplementary file 1 — Fig. S1 Cellular localization of heat shock 70 kDa protein 1 (HSPA1) in heart of mice 20 weeks after exposure to 16 Gy and 18 h after 1 h heat shock at 43 °C. Tissue was stained with FITC-labeled anti-HSPA1 antibody and counterstained with 4’,6-diamidino-2-phenylindole (DAPI) to visualize nuclei (representative pictures were registered at 100 × magnification). FITC fluorescein isothiocyanate [file 66_2017_1220_MOESM1_ESM.jpg]
